# Comprehensive Geriatric Assessment and quality of life after localized prostate cancer radiotherapy in elderly patients

**DOI:** 10.1371/journal.pone.0194173

**Published:** 2018-04-09

**Authors:** Aurore Goineau, Loïc Campion, Bénédicte d’Aillières, Brigitte Vié, Agnès Ghesquière, Guillaume Béra, Didier Jaffres, Guy de Laroche, Nicolas Magné, Xavier Artignan, Jérôme Chamois, Philippe Bergerot, Etienne Martin, Gilles Créhange, Elisabeth Deniaud-Alexandre, Xavier Buthaud, Yazid Belkacémi, Mélanie Doré, Laure de Decker, Stéphane Supiot

**Affiliations:** 1 Department of Radiation Oncology, Institut de Cancérologie de l’Ouest, Angers, France; 2 Department of Statistics, Institut de Cancérologie de l’Ouest, Saint Herblain, France; 3 Department of Radiation Oncology, Clinique Armoricaine de Radiologie, St Brieuc, France; 4 Department of Radiation Oncology, Centre Hospitalier de Bretagne Sud, Lorient, France; 5 Department of Radiation Oncology, Institut de Cancérologie de Loire, St Priest en Jarez, France; 6 Department of Radiation Oncology, CHP St Grégoire, St Grégoire, France; 7 Department of Radiation Oncology, Clinique Mutualiste de l’Estuaire, St Nazaire, France; 8 Department of Radiation Oncology, Centre Georges François Leclerc, Dijon, France; 9 Department of Radiation Oncology, Centre Hospitalier Départemental de Vendée, La Roche sur Yon, France; 10 Department of Radiation Oncology, Centre Catherine de Sienne, Nantes, France; 11 Department of Radiation Oncology, CHU Henri Mondor, Créteil, France; 12 Department of Radiation Oncology, Institut de Cancérologie de l’Ouest, Saint Herblain, France; Northwestern University, UNITED STATES

## Abstract

**Introduction:**

Radiotherapy can diminish quality of life (QoL) for prostate cancer patients. Our objective was to evaluate the effect of radiotherapy on QoL in men aged 75 years or older treated with radiotherapy for a localized prostate cancer, and to identify predictors of reduced QoL.

**Patients and methods:**

We prospectively administered a battery of geriatric (MNA, GDS, Get up and Go Test, CIRS-G, ADL, IADL, MMSE), toxicity (IPSS; IIEF 5), and QoL (QLQ C30) screening tests in 100 elderly patients before and two months after prostate cancer radiotherapy (NCT 02876237). Patients ≥ 75 years undergoing radiotherapy with a curative intent for localized prostate cancer with or without androgen deprivation therapy (ADL) were eligible for study inclusion. Correlations between patient-assessed QoL and tumor characteristics, radiotherapy treatment or CGA parameters were sought using the Fisher or the Mann and Whitney tests. Changes in QoL parameters over time were analyzed using the Wilcoxon signed-rank test.

**Results:**

At study entry, scores for IADL impairments were present in 51%, reduced autonomy in activities of daily living in 16%, cognitive impairment found in 20%, depression-related symptoms in 31%, and 66% of patients had significant co-morbidities. Eight percent were judged to be at risk of fall and 2% were found to be undernourished. Severely impaired (IPSS ≥ 20) urinary function was observed in 11.2% and 13.5% of patients before and two months after completion of radiotherapy respectively. Significantly decreased QoL (> 20 points) at two months after treatment was found in 13% of patients and a moderate but clinically relevant reduction (10 to 20 points) in 17% of patients. No tumor characteristic, treatment, or oncogeriatric parameter was predictive of reduced QoL following prostate cancer radiotherapy.

**Conclusion:**

Despite sometimes markedly diminished oncogeriatric parameters, prostate cancer radiotherapy was generally well tolerated in these elderly patients. We found no predictive factor to determine which patients would experience impaired quality of life following radiotherapy.

## Introduction

Prostate cancer mostly occurs in elderly men (aged ≥ 75 years) in whom it represents a major cause of impaired quality of life and is a leading cause of cancer mortality [1]. Localized prostate cancer in the elderly is mostly treated with androgen deprivation therapy (ADT) and/or radiotherapy, and surgery is only rarely recommended [2]. Although severe radiotherapy-induced toxicity is unusual in younger patients, occurring in fewer than 5% of them, curative radiotherapy is often believed to be less well tolerated in the elderly. Older patients tend to present with impaired urinary and digestive function that risk being by aggravated by radiotherapy. For this reason, only a minority of patients older than 75 with localized high-risk prostate cancer receive any local treatment. The majority receive palliative treatment (ADT or no therapy at all) [3], despite evidence showing significantly improved survival when radiotherapy is added to ADT [4,5].

The quality of life of patients undergoing intensity-modulated radiation therapy (IMRT) for prostate cancer often diminishes immediately after therapy [6,7], but most symptoms resolve within 6 months and long term quality of life is usually comparable to that prior to therapy [8–10]. Radiotherapy (and ADT) are particularly associated with increased asthenia and decreased social, physical and cognitive functioning at two months after treatment [6]. Retrospective studies suggest that prostate cancer radiotherapy is quite well tolerated in older patients [11,12], but despite the large burden of localized prostate cancer, prospective studies of quality of life after radiotherapy in this potentially fragile population are lacking.

The selection of elderly patients for risk-adapted oncology treatments remains a challenge for oncogeriatric study. Geriatric screening instruments offer one avenue of assistance to the clinician who wishes to orientate the fragile patient prior to any oncologic intervention [13]. Comprehensive Geriatric assessment (CGA) enables precise scoring of daily activities, risk of fall, undernutrition, depression, and comorbidities to help predict whether aggressive cancer treatments will be tolerated. Although CGA is largely used prior to medical interventions, such as chemotherapy [14] or ADT [15–17], specific studies of older prostate cancer patients undergoing radiotherapy are mostly retrospective, lack precise oncogeriatric evaluation [18–21] or focused on toxicity (assessed by physicians) and not on patient’s related quality of life [22].

We carefully analyzed the geriatric characteristics of a group of older patients and prospectively assessed toxicity and quality of life in order to be able to determine which factors might be predictive of poorly-tolerated radiotherapy.

## Patients and methods

We recruited patients with localized prostate cancer aged 75 or more for whom a multidisciplinary tumour board had recommended local prostate radiotherapy with curative intent, alone, or combined with ADT, to a prospective multicenter cohort study (NCT 02876237). Patients were recruited by radiation oncologists during the first consultation, after the multidisciplinary tumor board and before starting radiation therapy. Patients undergoing salvage prostate bed radiotherapy following surgery were also included. There was no exclusion criteria regarding general conditions. The study was approved by the appropriate ethics committees, the Comité Consultatif sur le Traitement de l’Information en matière de Recherche dans le domaine de la Santé (CCTIRS) and the Commission Nationale Informatique et Liberté (CNIL). All patients gave consent for participating to the collection and analysis of data of this study.

All patients underwent a complete comprehensive Geriatric Assessment (CGA) by a geriatrician prior to initiation of radiotherapy. The past medical, personal and social history, current medication, body mass index, and home to study center distance were documented. The Cumulative Illness Rating Scale for Geriatrics (CIRS-G) [23], Activities of Daily Life (ADL) [24] and Instrumental Activities of Daily Life (IADL) [25], Mini Mental State Examination (MMSE) [26], mini Geriatric Depression Scale (GDS) [27], Mini Nutritional Assessment (MNA) [28], and the get up and go test (GUAGT) [29] were performed and the scores recorded. See Annexe 1 for details.

Clinical target volumes and organ-at-risk volumes were determined according to the GETUG (Groupe d’Etude des Tumeurs Urogenital, french group for genito-urinary tumors study) recommendations for prostate [30], prostatic bed [31] and pelvic lymph node [32] contouring. Radiotherapy was delivered to a total dose of 74.8–80 Gy in 34 to 40 fractions to the prostate or 66 Gy in 33 fractions to the prostatic bed. In high-risk patients, pelvic lymph nodes received 46–54.4 Gy in 23 to 34 fractions. Hypofractioned radiotherapy to the prostate (60 Gy in 20 fractions) was allowed in accordance with the PROFIT trial guidelines [33]. Dose constraints for the rectum and the bladder followed the Quantec recommendations [34,35].

Patients with an intermediate prognosis according to D’Amico’s classification were eligible for 3–6 months of concurrent and adjuvant hormonal therapy, which was extended to 2 to 3 years in high risk patients [36]. ADT was to be administered prior to or on the first day of radiotherapy.

Patients completed the International Prostate Symptom Score (IPSS), the International Index of Erectile Function (IIEF-5), the core and prostate cancer–specific modules of the European Organization for Research and Treatment of Cancer (EORTC) quality of life (QoL) questionnaires (QLQ-C30 version 3.0) at the first consultation (before radiotherapy) and at two months after treatment. We did not monitor quality of life during radiotherapy because very different radiotherapy protocols were used (between 20 to 40 fractions in 4 and 8 weeks), instead focusing on early (2 months) assessment after radiotherapy. Items were combined according to EORTC criteria into several scales ranging from 1 to 100. The higher the score for global health and function, the better the QoL; the higher the score for symptoms, the poorer the QoL.

Correlations between patient-assessed QoL, their treatment, and CGA parameters were sought using the Fisher test for linear association (categoric variables) or the Mann and Whitney test (continuous variables). The significance threshold was p < 0.05, except for changes in QoL from baseline. A 10-point change in QoL was considered to be moderate but clinically relevant, severe for a 20-point change in score, and statistically significant if p < 0.01 [37,38]. These changes in QoL parameters over time (decrease on functional scoring and increase on symptoms scoring) were analyzed using the Wilcoxon signed-rank test.

## Results

### Assessment at study entry

One hundred patients presenting with intermediate (49%) or high-risk prostate cancer (48%) were recruited in 11 different cancer centers (Table 1). Their median age was 77.5 years (mean 78.4; range 75–89). The median distance between the patient’s home and radiotherapy center was 28 km (mean 32 km; range 4–147). The ADT was administered in 50% of patients. Severely (IPSS 20–35) or moderately impaired urinary function (IPSS 8–19) was observed in 11.2% (10/89) and 44.9% (40/89) of patients respectively. Among 78 patients who responded to the IIEF questionnaire which assesses sexual activity and erectile function, sexual activity was present in 26 patients (33.3%), among whom eight (10.3%) experienced normality or only minor dysfunction (IIEF 16–25), nine (11.5%) moderate dysfunction (IIEF 11–15), and nine (11.5%) severe dysfunction (IIEF 5–10). The CIRS-G revealed comorbidity in 66/100 patients, with 51/100 of patients presenting moderate to severe cardiovascular comorbidities. A GDS score ≥ 1 revealed depressive symptoms in 31/100 of patients. The activities of daily life (ADL score > 6) and Instrumental Activities of Daily Life (IADL score > 7) were impaired in 16/100 and 51/100 of patients respectively. Cognitive disorders (MMSE < 27) were revealed in 20/100 of patients. The calculated risk of fall (GUAGT <0) was 8/100 and undernutrition (MNA ≤17) was found in 2/100 of patients.

**Table 1:** Patient characteristic N = 100.

**Age**	%
75–79	72
80–85	24
≥ 85	2
**BMI**	
underweight (<18)	2
normal (18–25)	27
overweight (25–30)	50
obesity (>30)	21
**Number of medications**	
0–3	58
> 3	42
**Distance to radiotherapy center**	
< 30 km	54
30 à 60 km	27
≥ 60 km	19
**Clinical stage**	
low	3
Intermediate	49
high	48
**Geriatric problems**	
Depression	31
Impaired GUAGT	8
Denutrition	2
Comorbidities	66
ADL impairment	16
IADL impairment	51
Cognitive impairment	20
**Radiotherapy**	
prostate	76
prostate bed	24
**ADT**	
yes	50
no	50

BMI: body mass index

ADL: activities of daily life

IADL: instrumental activities of daily life

ADT: androgen deprivation therapy

### Assessment following radiotherapy

**Fig 1:**
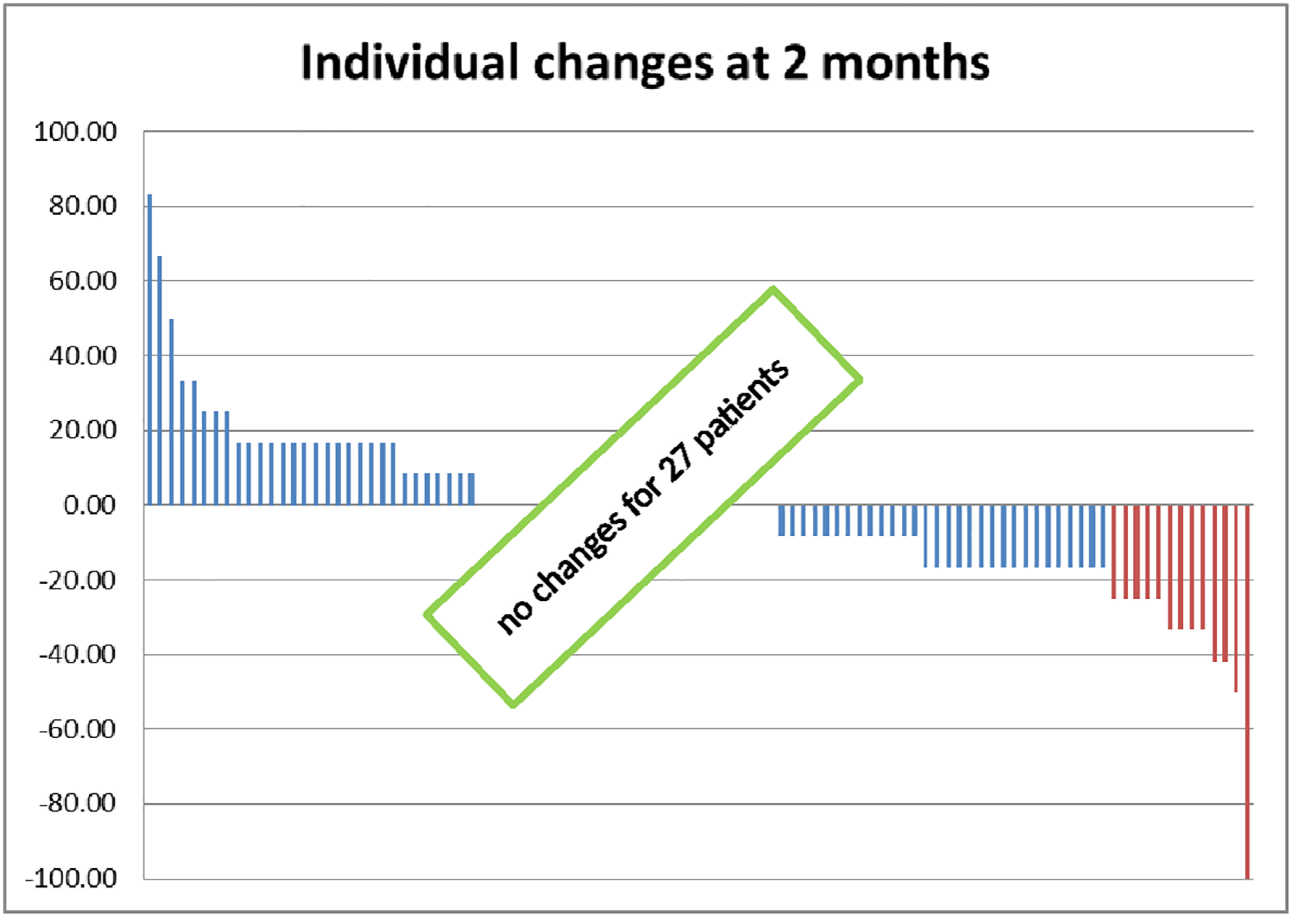
Individual changes in global QoL. Questions 29 and 30 in EORTC QLQ C30 questionnaires enable to calculated a score (from 0 to 100) for global QoL. Variations of this score before and 2 months after radiotherapy are reported here for each patient. There is an important decrease of QoL (≥ 20 points) for 13 patients and a moderate decrease in QoL (10 to 20 points) in 17 patients.

Two months after radiotherapy had concluded urinary function was assessed using the IPSS questionnaire in 89 patients. Severely (IPSS 20–35), moderately (IPSS 8–19), or only mildly impaired or normal function (IPSS <8) was noted in 13.5% (12/89), 44.9% (40/89) and 41.6% (37/89) of patients respectively. Similarly, among 71 patients who responded to a further IIEF questionnaire, sexual activity was maintained in 17 patients (23.9%). Among these, two (2.8%) experienced normal or only minor dysfunction (IIEF 16–25), four (5.6%) moderate impairment (IIEF 11–15) and eleven (15.5%) severe impairment (IIEF 5–10) of erectile function. We analyzed self-reported QoL following radiotherapy in 100 patients (see Fig 1 and Table 2). According to the core QoL questionnaire, general QoL was moderately decreased (loss of 10 to 20 points) in 17 patients (17%) and markedly decreased (loss ≥ 20 points) in another 13 patients (13%). On an individual basis (Table 2), the most frequent important (variation > 20 points) QoL alterations were diarrhea (21 patients), constipation (14), role functioning (20), fatigue (18) dyspnea (17) and insomnia (17).

**Table 2:** Variation of quality of life and symptoms parameters N = 100.

	Variation 10–20 (%)	Variation ≥ 20 (%)	Variation ≥ 10 (%)
**Global Health**	17	13	30
**Physical functioning**	5	9	14
**Role functioning**	12	20	32
**Emotional functioning**	7	4	11
**Cognitive functioning**	16	5	21
**Social functioning**	16	12	28
**Fatigue**	22	18	40
**Nausea / Vomiting**	1	3	4
**Pain**	12	11	23
**Dyspnoea**	0	17	17
**Insomnia**	0	17	17
**Appetite loss**	0	11	11
**Constipation**	0	14	14
**Diarrhoea**	0	21	21
**Financial difficulties**	0	4	4

**Fig 2:**
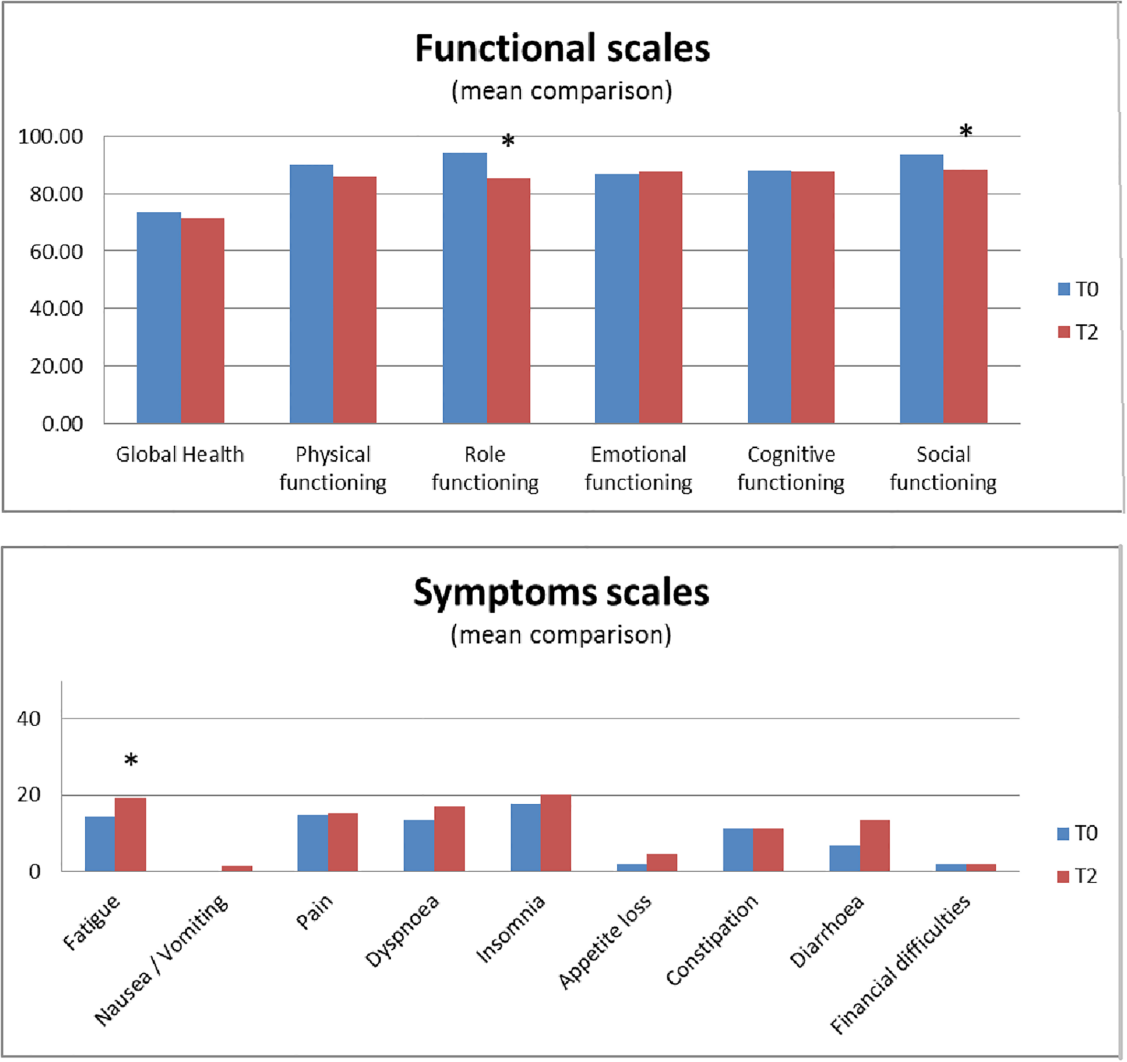
For each item of QLQ C30 questionnaire, we compare mean score before and 2 months after radiotherapy. For functional scales (Fig 2a), a high score is related to better QoL and for symptoms scores (Fig 2b), high score is related to high symptoms and worth QoL. Statistically significant variations.

We then analyzed mean score comparisons of each item on the QLQ C30 questionnaire between baseline and two months (Fig 2a and 2b). Significant (p<0.01) impairment in role (mean loss -9, p = 0.0001) and social functioning (mean loss -4.9, p = 0.0044) was observed at two months compared with baseline. On the symptom scales, only fatigue (mean increase +4.6, p = 0.0029) was significantly increased at two months.

### Predictive factors for impaired patient-reported outcomes

We wished to determine whether any clinical parameter or geriatric score was predictive for impaired QoL parameters (Table 3). No parameter was found significantly predictive for a deterioriation in general QoL. Better QoL at baseline was associated with more moderate alterations in QoL at two months after radiotherapy had concluded (p = 0.012).

**Table 3:** Predictive factors for moderate changes in quality of life.

	No QoL decrease > 10 points(n = 70)	QoL decrease > 10 points(n = 30)	p
Age	78 [75–89]	77 [75–88]	0.933
Distance	28 [4–147]	19 [5–85]	0.687
BMI	27.4 [20.5–42.9]	25.9 [19.8–34.9]	0.187
Number of medications	3 [0–6]	2 [0–6]	0.873
QLQ-C30 T0	75.0 [0–100]	83.3 [50.0–100]	0.012
Clinical stage			0.721
Low	3	0	
Intermediate	33	15	
High	34	15	
Radiotherapy			0.153
prostate	56	20	
prostatic bed	14	10	
ADT			0.614
No	33	16	
Yes	36	14	
Depression			0.422
No	50	19	
Yes	20	11	
Risk of fall			0.699
No	55	24	
Yes	5	3	
Denutrition			0.3
No	70	29	
Yes	0	1	
Comorbidities			1
No	24	10	
Yes	46	19	
Urinary symptoms			0.576
Light	25	14	
Moderate	29	11	
High	8	2	
ADL impairments			0.138
No	56	28	
Yes	14	2	
IADL impairments			1
No	34	15	
Yes	36	15	
Cognitive impairment			0.749
No	59	27	
Yes	10	3	
Urinary symptoms at 2 months			0.177
Mild	25	12	
Moderate	31	9	
Severe	6	6	

## Discussion

The decision as to whether to recommend local treatment for prostate cancer in the elderly remains challenging, and prospective data on the tolerability of radiotherapy in this population were lacking. Our comprehensive and systematic evaluation of a large representative cohort confirms that a substantial proportion of these patients are indeed at risk of fatigue and impaired QoL following radiotherapy. However, our most important finding is that despite numerous comorbidities and frequently impaired geriatric parameters, more than 70% of older patients undergoing radiotherapy maintain their overall QoL immediately after completion of radiotherapy. No geriatric, patient or treatment-related parameters were predictive of impaired QoL following radical radiotherapy.

The majority of these elderly patients presented with impaired geriatric parameters. More than half of our patients about to undergo radiotherapy had some pre-existing restriction in IADL. Moreover, two thirds of patients presented with comorbidities, especially cardiovascular comorbidities, which may affect ADT tolerance [36]. Unsuspected memory loss problems were discovered in one in five patients, and depression in almost one in three patients, which may also be aggravated by ADT [39]. These fragilities were usually found in metastatic cancer patients [40,41]. This underlines the utility of CGA prior to initiation of curative radiotherapy treatment, in order to provide appropriate overall care in this vulnerable population. The vulnerabilities of geriatric prostate cancer patients undergoing radiotherapy differ from those of metastatic patients undergoing chemotherapy [42]. Numerous studies have insisted upon the importance of nutritional status prior to drug administration, both for the tolerability of chemotherapy, and survival [43,44]. In our study, malnutrition was practically absent. Geriatric screening questionnaires place considerable weight on the evaluation of the nutritional status: on the G8 three questions out eight relate to nutrition [45]. Similarly impaired GUAGT is often sought by geriatricians [43] but was found in fewer than 10% of our patients. This suggests that a screening tool like G8 may be insufficient to fully evaluate the occult vulnerabilities in these elderly patients and that fuller CGA is needed, in accordance with other recent findings [22].

Despite impaired geriatric parameters, radiotherapy was well tolerated and the QoL maintained in a large proportion of patients. Urinary function was overall well preserved following radiotherapy (severe urinary symptoms in 11.2% of patients prior to radiotherapy compared with 13.5% after). This result contrasts with previous studies showing that increased age (>70) is a risk factor for increased urinary toxicity [46,47]. Longer follow-up is needed to fully assess long-term urinary toxicity. Important (> 20 points) acute diarrhea occurred in 21% of patients. Older patients are also at higher risk of digestive toxicity [48,49]. In a minority of patients (less than 20%), radiotherapy potentially increased fatigue, which consequently decreased social and leisure interactions. The source of fatigue during radiotherapy is multiple: comorbidities, transportation to the radiotherapy center, nocturia or ADT. Fatigue is very common in men with prostate cancer, particularly in those receiving ADT, and is a common side effect of prostate cancer radiotherapy [50,51], but whether this fatigue is greater in older patients remains unproven.

Despite acute toxicity, overall QoL was maintained or improved in 70% of patients and only 13% of patients complained of severely (> 20 points) decreased QoL at two months. These favourable results are similar to studies in younger patients, where radiotherapy combined with 6-months of androgen-depriving therapy did not profoundly alter physical or mental health as compared to active monitoring [52]. We previously evaluated longitudinal QoL following IMRT in a younger population (median age 73, range [50–80]), and found a similar pattern where quality of life was moderately impaired at two months but then returned to baseline after six months [6,9]. Other studies have also showed moderate but transient impaired QoL immediately after radiotherapy in younger patients [10,53,54]. However, more patients experienced moderately (17% vs 5.2%) or markedly (13% vs 10.5%) impaired overall QoL in this geriatric series than in our younger patient series. The impact of age on impairment of QoL remains an open question.

We hypothesized that a CGA prior to the initiation of radiotherapy, together with comprehensive collection of patient- and treatment-related data would enable us to predict decreased QoL at two months after radiotherapy had ended. Neither baseline oncogeriatric parameters, patient or tumor characteristics, distance to radiotherapy center, nor the use of ADT were correlated to severely (>20 points) or moderately (>10 points) impaired QoL. This might be explained by a quite limited sample and/or by the low number of patients experiencing decreased QoL. Some authors showed that higher levels of comorbidity diseases were predictive of decreased long-term global QoL following radiotherapy and ADT [55]. Others confirms our findings with difficult for CGA outcomes to predict significant acute radiotherapy toxicity [22]. We will pursue the analysis over a longer follow-up period and repeat CGA during follow up to evaluate the impact of geriatric characteristics on subsequent QoL following radiotherapy.

The generalizability of our study may be limited by a potential selection bias. In France, all patients who are candidates for radical radiotherapy treatment must be discussed at a multidisciplinary board meeting. During these meetings, the urologists often propose active monitoring or ADT for very fragile older patients. Considering the potential selection bias, we asked the investigators in each center. We can summarise as follow: about 5% of the patients we considered too frail for radiotherapy during multidisciplinary tumor board. Patients over 75 represent between 40% and 50% of all patients treated by radiotherapy for a localised prostate cancer. Successful recruitments were very different between the eleven centers, between 100% to 50%, and patient’s refusal was often due to important distances between home and radiotherapy center. Our patient population does not therefore reflect the general population of older men with prostate cancer. However, our study was not restricted to teaching hospitals (where tertiary referral may produce an atypical patient population) and more importantly, our CGA revealed that a large number of patients were considered to have CGA impairments. Our study is therefore reasonably representative of the population of patients whose life expectancy suggests that they may benefit from radical radiotherapy. Our study was restricted to acute toxicity and early evaluation of reported outcomes. We focused on an early time point (two months after treatment) because our previous study showed that QoL worsened immediately following radiotherapy but returned to baseline 18 months after completion of IMRT. However, delayed intestinal toxicity affecting QoL may occur years after completion of radiotherapy [52]. We therefore need to validate our results after longer follow-up. Another potential limitation of our study is that the CGA may have revealed treatable problems whose correction prior to radiotherapy may have reduced the impact of this treatment on QoL. To avoid this bias, a clinical trial randomizing CGA should be performed, as has been done in lung cancer [56].

## Conclusion

Localized prostate cancer of older men is a common clinical situation and the role of radical radiotherapy is controversial. Most patients are currently treated by active monitoring or ADT only. Our study is the first to extensively evaluate geriatric parameters prior to initiation of radiotherapy and longitudinally assess for QoL parameters following treatment. Our data suggest that QoL is mostly well conserved soon after radiotherapy, with or without ADT, even in this often debilitated patient population. This means that if there is a reasonable prospect that local radiotherapy will enhance the patient’s life expectancy, the fear of iatrogenic decreased QoL need not inhibit the decision to treat. An extensive CGA prior to initiation of radiotherapy can reveal comorbidities that may worsen after radiotherapy combined with ADT. We found no particular predictive factors for which older patients were most at risk of decreased QoL following radiotherapy. Further evaluation of long-term QoL is needed.

## Supporting information

S1 Dataset Full dataset for the study. (XLSX)

S1 Questionnaires Questionnaires and scoring (french version). (DOC)
