# Supplementary figures and images for: Comprehensive Geriatric Assessment and quality of life after localized prostate cancer radiotherapy in elderly patients
Source: PLoS One. 2018 Apr 9;13(4):e0194173. doi: 10.1371/journal.pone.0194173 (PMC5890970; doi:10.1371/journal.pone.0194173)

QLQC-30


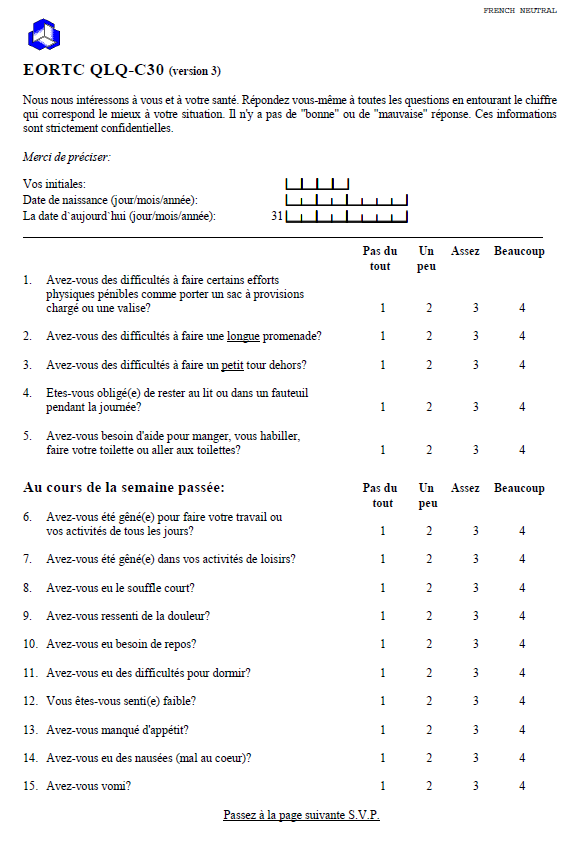


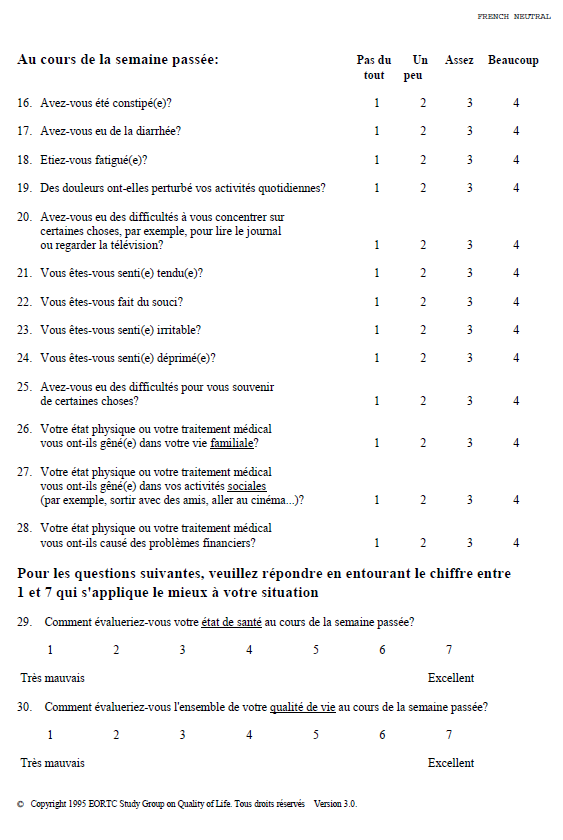


IPSS

IIEF 5


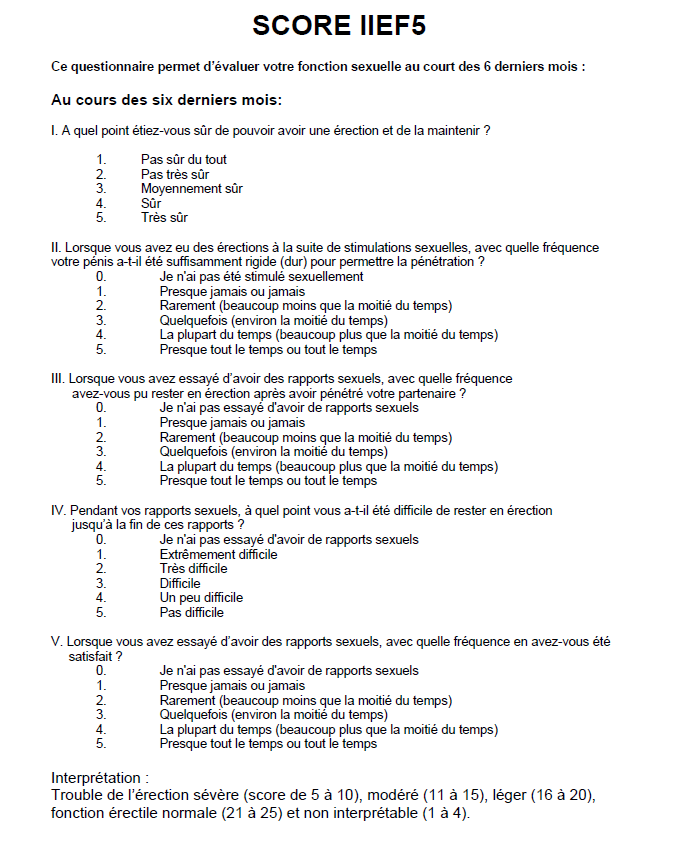


COMPREHENSIVE GERIATRIC ASSESSMENT


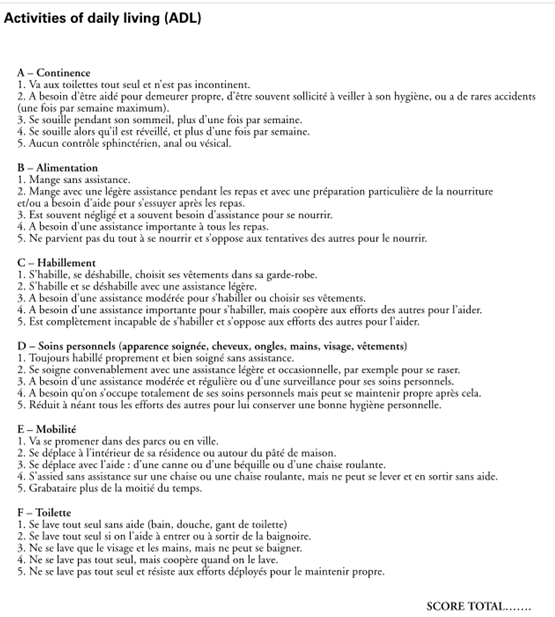


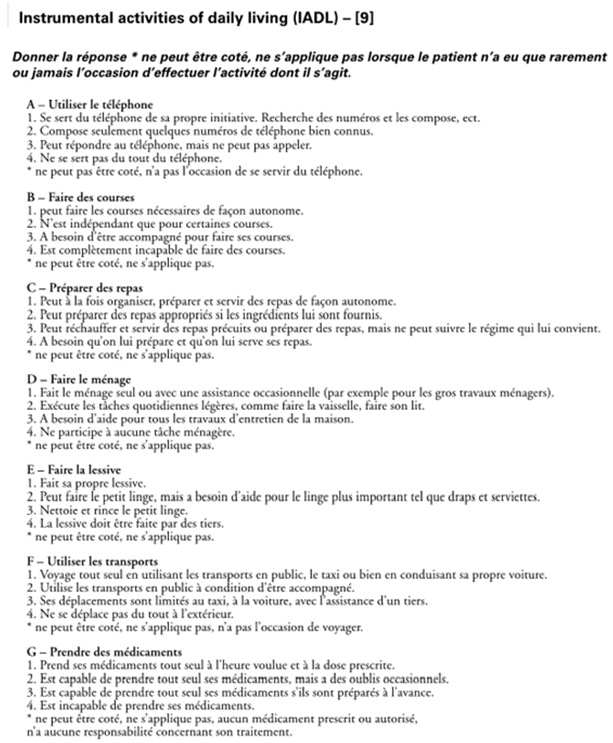


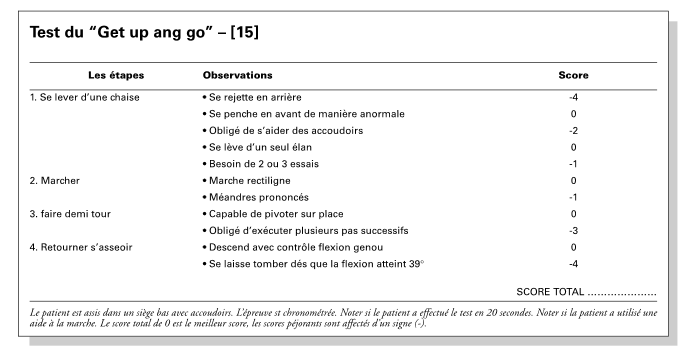

Supplement: S1 Questionnaires — (DOC) [file pone.0194173.s002.doc]
